# Combined cell-surface display- and secretion-based strategies for production of cellulosic ethanol with *Saccharomyces cerevisiae*

**DOI:** 10.1186/s13068-015-0344-6

**Published:** 2015-09-26

**Authors:** Zhuo Liu, Kentaro Inokuma, Shih-Hsin Ho, Riaan den Haan, Tomohisa Hasunuma, Willem H. van Zyl, Akihiko Kondo

**Affiliations:** Department of Chemical Science and Engineering, Graduate School of Engineering, Kobe University, 1-1 Rokkodai, Nada-ku, Kobe, 657-8501 Japan; Organization of Advanced Science and Technology, Kobe University, 1-1 Rokkodai, Nada-ku, Kobe, 657-8501 Japan; State Key Laboratory of Urban Water Resource and Environment, School of Municipal and Environmental Engineering, Harbin Institute of Technology, Harbin, 150090 People’s Republic of China; Department of Biotechnology, University of the Western Cape, Bellville, 7530 South Africa; Department of Microbiology, University of Stellenbosch, Stellenbosch, 7600 South Africa; Biomass Engineering Program, RIKEN, 1-7-22 Suehiro-cho, Tsurumi-ku, Yokohama, Kanagawa 230-0045 Japan

**Keywords:** Cell-surface display, Secretion, Cellulase, Ethanol, *Saccharomyces cerevisiae*

## Abstract

**Background:**

Engineering *Saccharomyces cerevisiae* to produce heterologous cellulases is considered as a promising strategy for production of bioethanol from lignocellulose. The production of cellulase is usually pursued by one of the two strategies: displaying enzyme on the cell surface or secreting enzyme into the medium. However, to our knowledge, the combination of the two strategies in a yeast strain has not been employed.

**Results:**

In this study, heterologous endoglucanase (EG) and cellobiohydrolase I (CBHI) were produced in a *β*-glucosidase displaying *S. cerevisiae* strain using cell-surface display, secretion, or a combined strategy. Strains EG-D-CBHI-D and EG-S-CBHI-S (with both enzymes displayed on the cell surface or with both enzymes secreted to the surrounding medium) showed higher ethanol production (2.9 and 2.6 g/L from 10 g/L phosphoric acid swollen cellulose, respectively), than strains EG-D-CBHI-S and EG-S-CBHI-D (with EG displayed on cell surface and CBHI secreted, or vice versa). After 3-cycle repeated-batch fermentation, the cellulose degradation ability of strain EG-D-CBHI-D remained 60 % of the 1st batch, at a level that was 1.7-fold higher than that of strain EG-S-CBHI-S.

**Conclusions:**

This work demonstrated that placing EG and CBHI in the same space (on the cell surface or in the medium) was favorable for amorphous cellulose-based ethanol fermentation. In addition, the cellulolytic yeast strain that produced enzymes by the cell-surface display strategy performed better in cell-recycle batch fermentation compared to strains producing enzymes via the secretion strategy.

**Electronic supplementary material:**

The online version of this article (doi:10.1186/s13068-015-0344-6) contains supplementary material, which is available to authorized users.

## Background

Due to the limitations in fossil fuel supplies and environmental issues, bioethanol derived from lignocellulosic materials has recently gained increased attention [1, 2]. *Saccharomyces cerevisiae* is the most commonly used microorganism for ethanol production, but lacks essential cellulolytic enzyme activities to degrade cellulose into glucose [3]. To resolve this problem, the construction of recombinant yeast strains capable of producing heterologous cellulases, including *β*-glucosidase (BGL), endoglucanase (EG), and cellobiohydrolase (CBH), has been pursued over the last two decades [4–6].

Currently, the production of cellulases follows two major strategies: displaying enzymes on the cell surface or secreting enzymes into the fermentation broth. The glycosylphosphatidylinositol (GPI) anchoring system enables the display of various kinds of enzymes on the cell surface [7]. The cell-surface display strategy increases the effective concentration of enzymes, and promotes a greater degree of synergy [8]. In addition, glucose liberated from cellulose in proximity to the cell surface is immediately taken up, thereby minimizing the risk of contamination or product inhibition [9]. Furthermore, immobilizing enzymes on the cell surface enables the re-use of enzymes and cells in multi-batch fermentations, which reduces the cost of yeast propagation and that of supplementation with extraneous enzymes [10, 11]. In contrast, secreting enzymes into the medium recreates the “free enzyme system”, which is similar to the cellulase system of filamentous fungi. The quantity of secreted enzymes is limited only by the production capacity of cells, not by physical restrictions, such as the incorporation capacity of yeast cell wall associated with cell-surface display [12]. Moreover, free cellulases can penetrate into the secondary cell walls of plant biomass [13], increasing the accessibility of cellulose, which was reported as the critical factor in enzymatic hydrolysis [14].

Thus, each strategy has both advantages and disadvantages. The selection of an optimal strategy for enzyme production should be based on the characteristics of a given enzyme and its reaction mechanism. It has been reported that cell-surface-displayed BGL exhibited higher efficiency in cellobiose usage than secreted BGL because of the improved stability caused by immobilization on the cell wall [15]. However, “display” systems may suffer from inefficiency of processive enzymes (e.g., CBH), leading to decreased hydrolysis efficiency compared to free enzyme systems [12]. Thus the combination of cell-surface display and secretion strategies into one recombinant yeast strain was expected to achieve improved hydrolysis of cellulose compared to either single strategy of displaying or secreting cellulases. Such a combined strategy may allow the various kinds of enzymes to be produced in their most appropriate location, assembling the advantages of the two strategies into one system of enzyme production.

Cellobiohydrolase I (CBHI) is the major component (~60 %) of the total cellulolytic protein of the cellulase system of *Trichoderma reesei* [16]. CBHI acts by hydrolyzing from the reducing end of crystalline cellulose fibers in a progressive manner. Recently, CBHI was reported as the main contributor to overall cellulose degradation, with other enzymes synergistically enhancing its hydrolytic efficiency [17]. Although CBHI has been heterologously expressed and secreted in *S. cerevisiae*, the relatively low titer [18, 19] and low specific activity [20] of secreted CBHI has limited the study of co-expression of CBHI with other cellulolytic enzymes. Recently, CBHI originating from *Talaromyces emersonii* fused with the *T. reesei* C-terminal carbohydrate-binding module (CBM) was efficiently expressed in *S. cerevisiae* with a yield of 100–200 mg/L, which is approximately 20-fold higher than the expression levels of *T. emersonii* CBHI reported elsewhere [21]. In separate work, immobilization of enzyme on the cell surface was reported to improve the stability of the enzyme [15]. However, to our knowledge, there has been no previous report of displaying CBHI on the cell surface of yeast strain.

In the present study, EG and CBHI were produced heterologously in a BGL-displaying *S. cerevisiae* strain using cell-surface display, secretion, or a combined strategy. The most suitable strategy for producing EG and CBHI for cellulose degradation was evaluated. Direct conversion of cellulose into ethanol was conducted by cellulolytic yeast strains and then applied in cell-recycle batch fermentation for further evaluation. To our knowledge, the work reported here is the first study on displaying CBHI on the yeast cell surface and the first study on the feasibility of combining the cell-surface display and secretion strategies in one yeast strain for heterologous cellulase production. We believe that this work will significantly increase our knowledge of how to engineer optimal yeast strains for biofuel production from cellulosic biomass.

## Results

### Construction of yeast strains

**Fig. 1:**
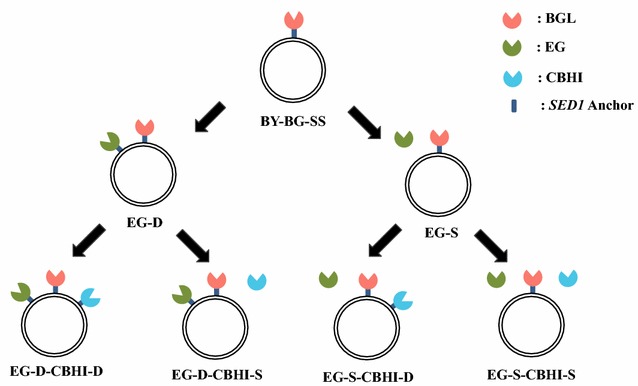
Schematic description of the recombinant yeasts strains constructed in this study

In this study, the haploid yeast strain *S. cerevisiae* BY4741 was used as the host strain for the heterologous expression of cellulase genes. The plasmids containing gene expression cassettes are listed in Table 1. All the gene expression cassettes included the *SED1* promoter and the *SAG1* terminator. The secretion signal peptide of BGL1 was derived from *Rhizopus oryzae* glucoamylase, while EGII and CBHI were produced with their native secretion signals. The GPI-anchoring region used for cell-surface display was constructed using the full length *S. cerevisiaeSED1* gene to display cellulases on the cell surface. It has been reported that the combination of *SED1* promoter and *SED1* anchoring region in a gene cassette enables highly efficient immobilization of enzyme into the cell wall [22]. Each cellulase gene was integrated into a separate internal open reading frame (ORF) region (I2 region for *EGII* gene and I5 region for *CBHI* gene) and confirmed by polymerase chain reaction (PCR). Figure 1 shows the cellulase production scheme of the recombinant yeast strains constructed in this study. Strain BY-BG-SS, which displayed *Aspergillus aculeatus* BGL1 on the cell surface, was reported previously [22]. The *T. reeseiEGII* gene expression cassettes, with and without the *SED1* anchoring region, were integrated into the genome of strain BY-BG-SS to yield strains EG-D and EG-S, respectively. Next, the expression cassettes of the *T. emersoniiCBHI* gene, with and without the *SED1* anchoring region, were integrated into the genome of strain EG-D to yield EG-D-CBHI-D and EG-D-CBHI-S, or into the genome of strain EG-S to yield EG-S-CBHI-D and EG-S-CBHI-S. The engineered yeast strains in this study are listed in Table 2.

**Table 1:** Characteristics of the integrative plasmids used in this study

Plasmid	Relevant features	References
pRDH225	*KanMX*, expression of *T. reesei* *EGII* gene	This study
pRDH226	*ZeoR*, expression of *T. emersonii CBHI* gene	This study
pIL2GA-SS	*LEU2*, display of *R. oryzae* glucoamylase	[50]
pIU5GA-SS	*URA3*, display of *R. oryzae* glucoamylase	[50]
pIBG-SS	*HIS3*, display of *A. aculeatus* BGL1	[22]
pIL2-EG_D_	*LEU2,* display of *T. reesei* EGII	This study
pIL2-EG_S_	*LEU2,* secretion of *T. reesei* EGII	This study
pIU5-CBHI_D_	*URA3,* display of *T. emersonii* CBHI	This study
pIU5-CBHI_S_	*URA3,* secretion of *T. emersonii* CBHI	This study

*R. oryzae*, *Rhizopus oryzae*; *A. aculeatus*, *Aspergillus aculeatus*; *T. reesei*, *Trichoderma reesei*; *T. emersonii*, *Talaromyces emersonii*; BGL1, *β*-glucosidase 1; EGII, endoglucanase II; CBHI, cellobiohydrolase I

**Table 2:** Characteristics of the *Saccharomyces cerevisiae* strains used in this study

Strains	Description	Expressed cellulases	Source
BY4741	*MAT* **a** *his3Δ1 leu2Δ0 met15Δ0 ura3Δ0*	–	Life Technologies
BY-BG-SS	BY4741 (pIBG-SS)	Display of BGL	[22]
EG-D	BY-BG-SS (pIL2-EG_D_)	Display of BGL, EG	This study
EG-S	BY-BG-SS (pIL2-EG_S_)	Display of BGL, secretion of EG	This study
EG-D-CBHI-D	EG-D (pIU5-CBHI_D_)	Display of BGL, EG, CBHI	This study
EG-D-CBHI-S	EG-D (pIU5-CBHI_S_)	Display of BGL, EG, secretion of CBHI	This study
EG-S-CBHI-D	EG-S (pIU5-CBHI_D_)	Display of BGL, CBHI, secretion of EG	This study
EG-S-CBHI-S	EG-S (pIU5-CBHI_S_)	Display of BGL, secretion of EG, CBHI	This study

In addition, the transcription levels of cellulase genes were determined after 72 h of cultivation. The transcription levels of the *BGL1*, *EGII*, and *CBHI* genes, each of which were under the control of a *SED1* promoter, were similar among all transformants (Additional file 1: Figure S1).

### Effect of multiple gene expression on cell growth

Cell growth was profiled to determine the metabolic burden caused by the expression of heterologous cellulase genes. Each of the engineered strains was inoculated into liquid YPD media and cultivated aerobically for 72 h at 30 °C. The host strain *S. cerevisiae* BY4741 was used as a reference strain. As shown in Additional file 2: Figure S2, no apparent difference in cell growth was observed between the host strain and any of the recombinant yeast strains.

### Direct ethanol production from cellulosic materials

Barley *β*-glucan and phosphoric acid swollen cellulose (PASC) were utilized as fermentation substrates. *β*-Glucan is a linear, water-soluble polysaccharide composed of 6 or 7 *β*-1,4-linked glucose residues [23]. PASC, which is derived from phosphoric acid treatment of Avicel PH-101, is an insoluble cellulosic material with more amorphous regions and a lower degree of crystallinity compared to Avicel [24, 25].

**Fig. 2:**
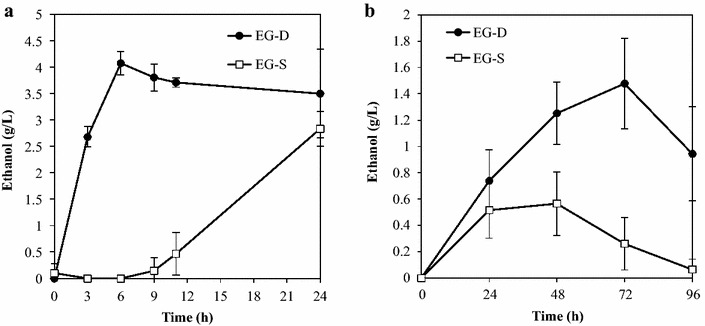
Time course of direct ethanol production from cellulosic materials by recombinant strains EG-D and EG-S. **a** Ethanol production from *β*-glucan. **b** Ethanol production from PASC. For each strain and time point, data are presented as the mean ± SD from three independent experiments

As depicted in Fig. 2a, ethanol production from 10 g/L *β*-glucan was performed using strains EG-D and EG-S. Yeast strains were cultivated in YPD medium for 72 h; cells then were collected by centrifugation and inoculated into fermentation medium at an initial cell concentration of 50 g wet cells/L. The ethanol fermentation was conducted under oxygen-limited conditions at 37 °C for 24 h. Ethanol production by strain EG-D initiated immediately after the start of fermentation and reached a maximum of 4.1 g/L after 6 h of fermentation. In contrast, strain EG-S exhibited a long lag phase before the start of ethanol production; no ethanol was detected until 9 h of fermentation.

The fermentation abilities of EG-D and EG-S also were evaluated by performing direct ethanol production from 10 g/L PASC (Fig. 2b). The fermentation was conducted under oxygen-limited conditions at 37 °C for 96 h with an initial cell concentration of 150 g wet cells/L. Ethanol production by strain EG-D peaked at 1.5 g/L ethanol at 72 h, while the production by strain EG-S peaked at 0.6 g/L at 48 h. These results revealed that locating EG on the cell surface improved the ethanol production from both soluble and insoluble cellulosic materials.

**Fig. 3:**
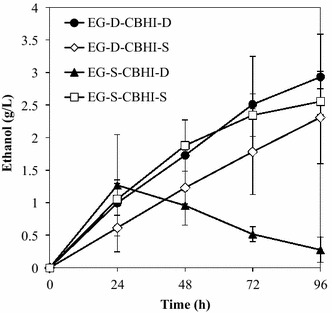
Time course of direct ethanol production from PASC by recombinant strains EG-D-CBHI-D, EG-D-CBHI-S, EG-S-CBHI-D, and EG-S-CBHI-S. For each strain and time point, data are presented as the mean ± SD from three independent experiments

To investigate the most suitable strategy for EG and CBHI production, direct ethanol production from 10 g/L PASC was evaluated using recombinant strains EG-D-CBHI-D, EG-D-CBHI-S, EG-S-CBHI-D, and EG-S-CBHI-S. As shown in Fig. 3, ethanol production by strains EG-D-CBHI-D and EG-S-CBHI-S peaked at 2.9 and 2.6 g/L (respectively) after 96 h of fermentation. Ethanol production by strain EG-D-CBHI-S peaked at 2.3 g/L at 96 h while strain EG-S-CBHI-D peaked at 1.2 g/L at 24 h. To further characterize the fermentation capacity of our constructs, the strains were compared by evaluating the PASCase and individual cellulase enzyme activities in PASC fermentation, and by testing the strains in cell-recycle batch fermentation.

### Enzyme activity in direct ethanol production from PASC

**Fig. 4:**
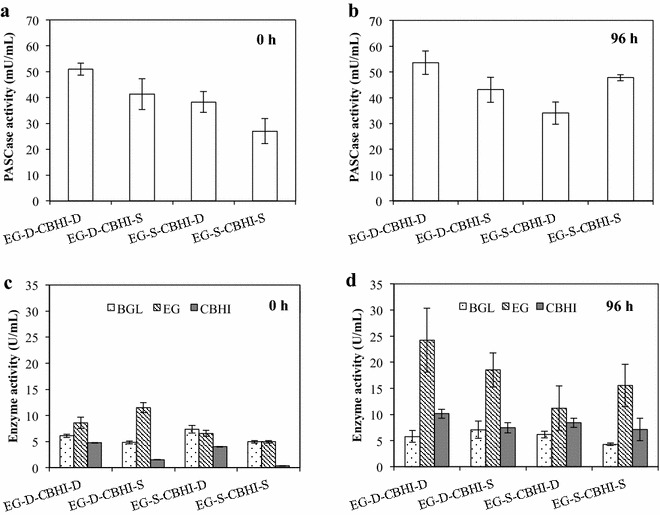
PASCase activities and cellulase activities of the cellulolytic *S. cerevisiae* strains in PASC fermentation. **a** PASCase activity at 0 h of the fermentation. **b** PASCase activity at 96 h of the fermentation. **c** Cellulase activity at 0 h of the fermentation. **d** Cellulase activity at 96 h of the fermentation. For each strain and time point, data are presented as the mean ± SD from three independent experiments

The cellulose degradation ability of cellulolytic strains is considered as one of the critical factors in the conversion of cellulose into ethanol. In this study, PASCase activity represents the PASC degradation capability of the cellulolytic yeast strains. The PASCase activity at 0 and 96 h of ethanol production from 10 g/L PASC was investigated. As shown in Fig. 4a, b, PASCase activity was highest (among the four recombinant yeast strains) in EG-D-CBHI-D at both 0 h (50.9 mU/mL) and 96 h (53.6 mU/mL). The PASCase activity of strain EG-S-CBHI-S increased from 26.9 mU/mL at 0 h to 47.8 mU/mL at 96 h. By contrast, the PASCase activity of strain EG-S-CBHI-D decreased after 96 h of fermentation, exhibiting the lowest activity (34.1 mU/mL) compared with other recombinant yeast strains.

The activity of BGL, EG, and CBHI from yeast strains at 0 and 96 h of PASC fermentation also was determined, as illustrated in Fig. 4c, d. BGL activity was similar among the four recombinant strains and remained similar (within a given strain and among different strains) during the fermentation. EG activity at 96 h was highest in strain EG-D-CBHI-D (24.2 U/mL) when compared with that in the other cellulolytic yeast strains. Initial (0 h) CBHI activity was highest in strain EG-D-CBHI-D (4.7 U/mL), and lowest in strain EG-S-CBHI-S. After 96 h of fermentation, CBHI levels appeared to rise similarly in the four strains, achieving activities of ~7–10 U/mL.

### Cell-recycle batch fermentation

**Fig. 5:**
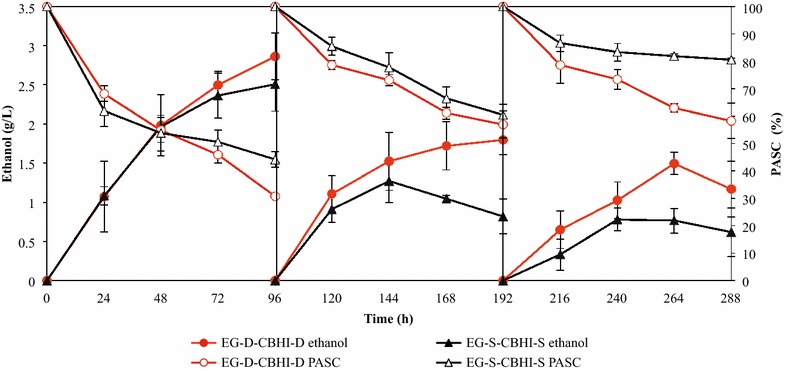
Three cycles of CRBF using recombinant *S. cerevisiae* strains EG-D-CBHI-D and EG-S-CBHI-S.* PASC (%)* indicates the percentage of residual PASC normalized to the initial concentration in the respective cycle. The initial amount of PASC in each cycle was defined as 100 %. For each strain and time point, data are presented as the mean ± SD from three independent experiments

To investigate the efficiency of strains EG-D-CBHI-D and EG-S-CBHI-S in a continuous process, cell-recycle fermentation was conducted under anaerobic conditions (Fig. 5). Recombinant yeast cells were collected for recycling and 10 g/L PASC was added into the fermentation medium at the beginning of each run. In the first batch, 69.3 and 55.9 % of PASC (corresponding to 6.9 and 5.6 g/L PASC) was converted into 2.9 and 2.5 g/L ethanol by strain EG-D-CBHI-D and strain EG-S-CBHI-S, respectively. In the 3rd batch, 41.8 % of PASC was consumed by strain EG-D-CBHI-D, corresponding to ~60 % of the consumption in the 1st batch. In contrast, the consumption of PASC in strain EG-S-CBHI-S decreased from 55.9 to 19.4 % after 3-cycle repeated fermentation. Correspondingly, the final ethanol titer generated by strain EG-D-CBHI-D was 1.7-fold higher than that generated by strain EG-S-CBHI-S in the 3rd batch. These results suggested that associating EG and CBHI with cells facilitated retention of cellulolytic activity even after three cell-recycles.

## Discussion

In this study, we integrated heterologous *EG* and *CBHI* genes into the genome of a BGL-displaying *S. cerevisiae*, permitting the production of EG and CBHI via cell-surface display, secretion, or a combined strategy. The recombinant strains that produced EG and CBHI in the same space (on the cell surface or in the medium) showed superior performance in the production of cellulosic ethanol. To our knowledge, this is the first report on combining cell-surface display and secretion strategies in a single yeast strain for heterologous cellulase production.

The benefits of attaching BGL to the yeast cell wall have been reported previously [15]. However, suitable strategies for EG and CBH production by cells remained obscure. In the present study, direct ethanol production from *β*-glucan and PASC was performed using cellulolytic yeast strains. Specifically, the production of ethanol was compared to investigate the suitable strategy for producing various kinds of cellulases. When *β*-glucan was used as the fermentation substrate, the ethanol production rate of strain EG-D was apparently higher than that of EG-S. Notably, strain EG-D was able to convert *β*-glucan into ethanol immediately after the start of fermentation, indicating that the displayed BGL and EG were successfully transferred into the fermentation medium with the cell inoculum. In contrast, strain EG-S converted *β*-glucan into ethanol after a 9-h lag, consistent with the need for this strain to accumulate (via secretion) EG in the medium following inoculation. This phenomenon demonstrated the advantage of an EG-display system to the cellulose fermentation process, since display permitted early onset of the production of ethanol. Compared with *β*-glucan, PASC contains a higher degree of polymerization and crystallization, rendering this substrate more difficult to degrade by BGL and EG. Indeed, fermentation with PASC yielded apparently lower rates of ethanol production than those seen upon fermentation with *β*-glucan. The decline of ethanol observed in PASC fermentation is probably due to the consumption via yeast cells when cellulases could not hydrolyze sufficient glucose from PASC. Notably, the rate of ethanol production from PASC by EG-D appeared higher than that by EG-S.

Subsequently, the *CBHI* gene was successfully expressed along with *BGL* and *EG* genes in *S. cerevisiae*. The *T. emersonii* CBHI used in this study was fused with the CBM of *T. reesei* CBH1 [21]; use of the CBM has been shown to enhance the adsorption of enzyme to its substrate and modify substrate surfaces to facilitate enzymatic hydrolysis [26]. We observed that co-production of CBHI with displayed EG enhanced ethanol production by up to twofold, demonstrating that the heterologous CBHI that was present assisted in the degradation of PASC. In the display strategy, the CBHI gene was expressed via the *SED1* expression cassette and anchored on the cell surface of *S. cerevisiae* using the SED1 anchoring domain [22]. It has been reported that the expression level of the *SED1* gene was highly induced in the stationary phase by various environmental stresses, such as ethanol [27]. In the present work, expression of both *CBHI* and *EG* gene by cell-surface expression cassettes (in strain EG-D-CBHI-D) permitted a doubling of CBHI activity at 96 vs. 0 h of fermentation, confirming the utility of the stress-induced *SED1* expression cassette. However, we note that the SED1 anchoring domain was fused to the C-terminus of the CBHI-CBM chimera protein; N- and C-terminal of CBM were fused with CBHI and SED1 anchoring domain, respectively, which may hinder the function of the CBM. Alternatively, an N-terminal anchoring domain, such as the N terminus flocculation functional domain of Flo1p, may be more suitable for displaying the chimeric CBHI containing a C-terminal CBM [7]. Nonetheless, to our knowledge, the present work represents the first report on displaying a CBHI on the cell surface of yeast strain.

**Fig. 6:**
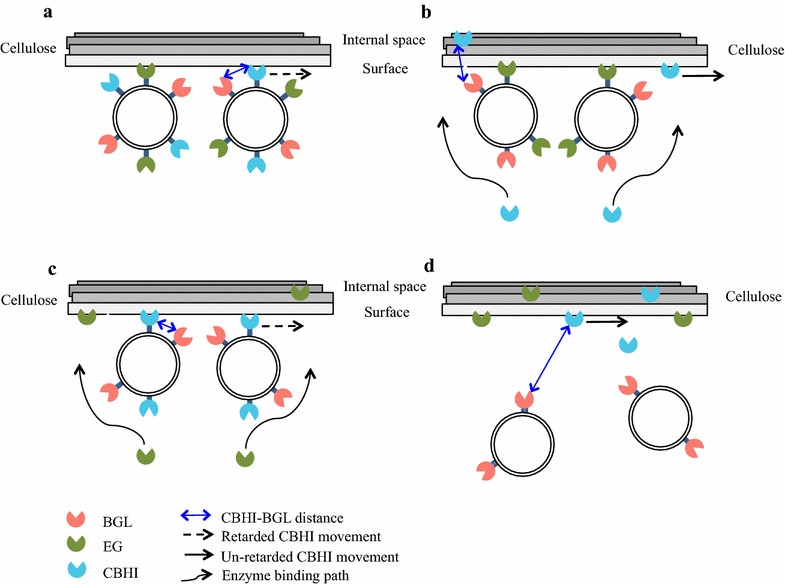
The effect of different locations of EG and CBHI on the conversion of cellulose into ethanol. **a** EG-D-CBHI-D. **b** EG-D-CBHI-S. **c** EG-S-CBHI-D. **d** EG-S-CBHI-S. The diagrams suggest the multiple factors involved in the degradation of cellulose, such as the distance between synergistic enzymes (CBHI-BGL distance), the effect of cell-surface display on the processive movement of CBHI (retarded/un-retarded CBHI movement), and the steric restriction during the binding of cellulases to cellulose surface (enzyme binding path)

In a previous report, a yeast strain displaying BGL, EG, and cellobiohydrolase II (CBHII) on the cell surface yielded higher ethanol production than a strain secreting the corresponding enzymes in free form [28]. Immobilized BGL on the cell wall is considered more appropriate for cellulosic ethanol production compared to free BGL [15]; our use of cell-surface-displayed BGL is presumably one of the reasons for the elevated ethanol yields in the present study. Ethanol production by strain EG-S-CBHI-S was similar to that of strain EG-D-CBHI-D. To understand this interesting result, the mechanism of the enzymatic hydrolysis of cellulose should be taken into account. EG can randomly cleave the amorphous regions of cellulose to produce oligosaccharides and provide free chain ends for CBH activity. Then CBHI can initialize cleavage from the free chain ends, degrading crystalline cellulose into cellobioses in a processive manner [29]. The binding of EG and CBHI onto the cellulose surface, along with the processive movement of CBHI, are considered essential steps in the degradation of cellulose [29]. Besides enzymes, the presence of a living microorganism is also important to the hydrolysis of cellulose. Generally, the radius of a spherical yeast cell is around 2 μm [30], which is nearly 400-fold larger than the hydrodynamic radius of a cellulase protein (≥5 nm) [31]. The immobilization of enzymes onto the cell wall may block the movement of processive enzymes (e.g., CBHI) or cause steric restriction in the collision with cellulose. As shown in Fig. 6a, in the case of strain EG-D-CBHI-D, three different cellulases were displayed on the cell surface in relatively close proximity (e.g., CBHI-BGL distance) compared to the strains constructed by other strategies. Such co-localization is expected to increase the occurrence of synergistic interactions among cellulases [8] and to facilitate the transportation of glucose into cells. However, due to the size of the cell–enzyme complex, the penetration of EG and CBHI into the internal space of cellulose is expected to be limited; such penetration has been reported as an important determinant of hydrolytic rate [14, 32]. Additionally, the processive movement of CBHI may be retarded due to its immobilization on the cell. In previous pre-steady-state analyses of CBHI activity on cellulose, stalling of the processive movement of CBHI was reported to lead to lower specific activity [33, 34]. By contrast, EG and CBHI in strain EG-S-CBHI-S were produced as free forms (Fig. 6d), a strategy that was expected to decrease steric hindrance and to increase the chance of collision with the substrate. Although the cellulases appeared to accumulate in fermentation with EG-S-CBHI-S (rising from 27 mU/mL at 0 h to 48 mU/mL at 96 h), the cellulolytic enzyme activity was still lower than that of EG-D-CBHI-D. Additionally, the diffusion efficiency of enzymes might affect the ability to degrade cellulose, especially in a substrate with higher viscosity (e.g., PASC). Thus, co-locating EG and CBHI in the same space (on the cell surface or in the medium) is favorable for amorphous cellulose-based ethanol fermentation.

By contrast, ethanol production levels were apparently lower in strains EG-D-CBHI-S and EG-S-CBHI-D. We infer that yeast cells attach to the surface of cellulose to facilitate the collision between immobilized cellulases and cellulose, but that this proximity may block the access of secreted EG or CBHI to the surface of the substrate (Fig. 6b, c). For instance, the cellulase activities of strain EG-D-CBHI-S at 96 h were higher than those of EG-S-CBHI-S, while PASCase activity was lower than that of strain EG-S-CBHI-S, effects that may be due to the steric hindrance mentioned above. Additionally, the hydrolysis efficiency of strain EG-S-CBHI-D appeared to be further decreased, presumably by the retarded movement of immobilized CBHI on the cell surface, as evidenced by the lower PASCase activity and ethanol production obtained in strain EG-S-CBHI-D. Interestingly, the individual enzyme activities in strain EG-D-CBHI-D were raised while PASCase activity kept constant, assuming that only the enzymes located in the contact region between yeast cell and cellulose could participate in cellulose degradation. Although the individual enzyme activities in whole cell were raised, the improvement of cellulolytic activity in “attaching region” is limiting. In contrast, most of the free cellulases can bind to cellulose and then participate in hydrolysis; the increase of individual enzyme activities could be reflected on improvement of cellulolytic activity. This may also explain why the overall enzyme activity of strain EG-S-CBHI-S (secretion system) rose observably (about 77 %) along with the increase of individual enzyme activities. Nonetheless, our results do indicate that the involvement of the microorganism in the synergism of cellulases may affect their hydrolysis efficiency towards cellulose. Moreover, the interactions between microbial cells and cellulose may also affect the cellulose degradation process. For instance, Francisco et al. reported that the engineered *E. coli* with displayed Cex CBH on the surface exhibited specific adhesion capacity toward cellulose [35], suggesting that the cell-to-cellulose adhesion pattern may lead to a distinct mechanism from that of free cellulases to break down cellulose.

From an industrial point of view, the reuse of yeast cells through various rounds of fermentation may be important in the ethanol production process [36]. Cell-recycle batch fermentation (CRBF) is a semi-continuous operation strategy using high densities of recycled cells to produce ethanol continuously. In previous reports, the CRBF of lignocelluloses was performed by the addition of large amount of commercial cellulases, a step that represents one of the main bottlenecks for commercialization [37, 38]. The application of a cellulolytic yeast strains to CRBF is expected to decrease the need for the addition of costly commercial enzymes [4]. Thus, in this study, the direct ethanol production from PASC using recycled cellulolytic cells was conducted without the addition of commercial cellulases. After a 3-batch recycle fermentation, our strain EG-D-CBHI-D retained 60 % of the PASC degradation ability that the strain had in the 1st batch. This retention of activity was 1.7-fold higher than that seen in strain EG-S-CBHI-S. The apparently lower degradation ability in strain EG-S-CBHI-S likely can be attributed to the loss of cellulases in each cycle, as most secreted enzymes were separated from cells during the cell collection step at the beginning of each batch. Although the amount of enzyme produced was sufficient for saccharification in the first batch, the ability to generate cellulases in each new cycle apparently declined in subsequent batches. In contrast, the enzymes immobilized on strain EG-D-CBHI-D showed more consistent activity than the activities in strain EG-S-CBHI-S during the recycling. Khaw et al. reported that the ethanol production rate of a yeast strain producing displayed α-amylase was maintained during a number of repetitions [39]. Consequently, the construction of cellulolytic yeast by cell-surface display strategy is more applicable to CRBF compared with the secretion strategy. Matano et al. even reported that with the addition of extraneous cellulases (10 FPU/g-biomass), the fermentation ability of a cellulase-displaying yeast strain remained constant after 5-cycle repeated-batch fermentation [40]. However, the required amount of additional cellulase was still too high, precluding economic feasibility for an industrial process [40]. In future work, we propose to further improve the cellulolytic activity of yeast strains and apply the cell-surface display strategy to ethanol production from lignocellulosic biomass (e.g., rice straw) with addition of trace amounts of exogenous cellulases.

## Conclusions

In this study, we investigated suitable strategies for heterologous production of EG and CBHI production in the engineering of cellulolytic *S. cerevisiae* strains. We demonstrated that cell-surface display system enhanced the production rate of ethanol. Placement of EG and CBHI in the same space (both on the cell surface or both secreted into the medium) was favorable for ethanol production from amorphous cellulose. In addition, a cellulolytic yeast strain producing cellulases via cell-surface display was more effective in the cell-recycle batch fermentation than a strain producing secreted cellulases.

## Methods

### Microbial strains and media

The relevant features and sources of the yeast strains used in this study are listed in Table 2. Strain *Escherichia coli* NovaBlue (Novagen, Inc., Madison, WI, USA) was used for the propagation of the plasmids. Bacterial cells were grown at 37 °C in Luria–Bertani broth (10 g/L tryptone, 5 g/L yeast extract, and 5 g/L sodium chloride) containing 100 mg/L ampicillin. Haploid yeast *S. cerevisiae* BY4741 (Life Technologies, Carlsbad, CA, USA) was used for cellulase production. Yeast strains were screened and pre-cultivated in synthetic dextrose (SD) medium [6.7 g/L of yeast nitrogen base without amino acids (Difco Laboratories, Detroit, MI, USA) and 20 g/L of glucose] supplemented with appropriate amino acids in a shaker incubator (150 rpm) at 30 °C, and then aerobically cultivated at 30 °C in YPD medium [20 g/L peptone (Bacto-Peptone™, Difco Laboratories), 10 g/L yeast extract and 20 g/L glucose]. Ethanol fermentation was performed in YP medium (10 g/L yeast extract and 20 g/L peptone) containing either 10 g/L PASC or 10 g/L *β*-glucan from barley (Megazyme, Bray, Ireland). PASC was prepared from Avicel PH-101 (Fluka Chemie GmbH, Buchs, Switzerland), as previously described [6].

### Plasmid and strain construction

The plasmids and primers used in this study are summarized in Table 1 and Additional file 3: Table S1, respectively. To construct the plasmid pRDH225, the gene *T. reesei EGII* was cloned as a 1277 bp *Pac*I/*Asc*I fragment from pRDH147 [41] into pBZD2 [42] to form pRDH225. To construct pRDH226, the *T. emersonii CBHI*-encoding gene containing a domain encoding a carboxy-terminal CBM originating from the *T. reesei CBHI* was amplified using Phusion hi-fidelity polymerase (Thermo Scientific) as directed by the manufacturer from pMI529 [21] as template with primers TeCBH1-L and TeCBH1-R. The resulting 1567 bp fragment was cloned as a *Pac*I/*Asc*I fragment into pBZD2 to form pRDH226.

The integrative plasmids for cell-surface display with the *SED1* anchor were constructed as follows: the DNA fragment encoding EGII from *T. reesei* was amplified from plasmid pRDH225 by PCR using the primers TrEG2-F and TrEG2-R. The cell-surface display cassette, which includes the I2 region (the 3′ non-coding region between gene *YFL021W* and *YFL020C*, used for integration), *LEU2*, *SED1* promoter, *SED1* anchoring region, and *SAG1* terminator was amplified from plasmid pIL2GA-SS using primers P-EG2 and EG2-A. Two DNA fragments were connected by the isothermal assembly method [43], generating the plasmid pIL2-EG_D_. To construct the secretion expression cassette without the *SED1* anchoring region, amplification was performed using the plasmid pIL2GA-SS as a template with primers P-EG2 and EG2-T. Primers TrEG2-F and TrEG2-R2 were used for amplifying the fragment encoding EG. The resulting plasmid from the combination of two DNA fragments was named pIL2-EG_S_.

The construction of CBHI integrative plasmids was performed by a process similar to the above description. For the cell-surface display plasmid, the DNA fragment of CBHI from *T. emersonii* was amplified from plasmid pRDH226 using primers TeCBHI-F and TeCBHI-R, and fused with the PCR product amplified from plasmid pIU5GA-SS by primers P-CBHI and CBHI-A. The resulting plasmid, which was named pIU5-CBHI_D_, can integrate into I5 region (the 3′ non-coding region of gene *YLL055W* and *YLL054C*). Plasmid pIU5-CBHI_S_ is the integrative plasmid with the secretion expression cassette, connected by the segment of CBHI-encoding gene (primes TeCBHI-F and TeCBHI-R2) and the PCR products amplified from plasmid pIU5GA-SS (primers P-CBHI and CBHI-T for secretion expression cassette).

Plasmids were transformed into *S. cerevisiae* BY4741 using lithium acetate as described [44]. The transformants were identified using colony PCR to check the integration of the cellulase gene expression cassettes (primers I2-F and I2-R for I2 insertion of EG-encoding cassette, and primers I5-F and I5-R for I5 insertion of CBHI-encoding cassette). Transformants with one copy of the cassette were selected for subsequent experiments.

### Quantification of the transcription level of cellulase-encoding genes by real-time PCR

The transcription levels of the cellulase-encoding genes were quantified by real-time PCR as described previously [45]. The PCR primers BGL 761F and BGL 858R [46] were used to determine the transcription level of gene *BGL1*. Primers rt-EG-F and rt-EG-R were used for the *EGII* gene and primers rt-CBHI-F and rt-CBHI-R were used for the *CBHI* gene. Transcription levels of the target genes were normalized to the housekeeping gene *ACT1* (primers rt-ACT1-R and rt-ACT1-F).

### Yeast cell growth assay

To measure cell growth, the parent strain and the engineered strains were cultivated individually in SD medium at 150 rpm for 24 h at 30 °C. The pre-cultured medium was inoculated into 5 mL YPD medium in a L-shaped vitreous tube at the initial OD_660_ of 0.05 and cultivated at 30 °C. The value of the OD_660_ was measured once hourly using a TVS062CA Bio-photorecorder (Advantec Toyo, Tokyo, Japan). The value of the OD_660_ was taken as an indicator of cell growth.

### Ethanolic fermentation

Recombinant yeast strains were pre-cultivated in SD medium for 24 h, then inoculated into YPD medium and aerobically cultured in YPD medium at 30 °C for 72 h. Cells were harvested by centrifugation at 3000×*g* for 10 min at 4 °C, and then washed twice with sterile distilled water. The wet cell pellet was weighed and then resuspended in 20 mL YP medium containing 10 g/L PASC or *β*-glucan from barley at an initial cell concentration of 150 g wet cells/L (PASC) or 50 g wet cells/L (*β*-glucan). Ethanol fermentation was performed at 37 °C for 96 h with 200 rpm agitation in 100 mL closed bottles, each equipped with a siliconized tube and check valve (Sanplatec Corp., Osaka, Japan) as a CO_2_ outlet under the oxygen-limited conditions. The ethanol concentration in the fermentation medium was determined using a gas chromatograph (model GC-2010; Shimadzu, Kyoto, Japan), as described previously [47].

In the cell-recycle batch fermentation, after the 96-h batch fermentation described above, cells were collected by centrifugation at 8000×*g* for 10 min at 4 °C. The pelleted cells were inoculated into fresh YP medium supplemented with 10 g/L PASC. The fermentation was repeated three times sequentially under the oxygen-limited conditions.

To measure PASC amount in fermentation, the fermentation broth (including the cells and residual PASC) was sterilized at 121 °C, 20 min (to terminate glucose consumption by yeast cells) and then cooled to room temperature. Sterilized medium was incubated with 3 FPU/g PASC commercial cellulase (Cellic CTec2; Novozymes Inc., Bagsvaerd, Denmark) for 2 h at 50 °C. After the hydrolysis reaction, the supernatant was obtained by centrifugation at 8000×*g*, 10 min, 4 °C. Glucose concentration in the supernatant was measured by the Glucose CII kit (Wako Pure Chemical Industries, Ltd., Osaka, Japan) and taken as the amount of PASC remnant.

### Enzyme assay

At the 0 and 96-h time points of ethanol fermentation, fermentation medium was assayed for PASCase, and individual cellulase activities. PASCase activity represents the PASC degradation ability of all enzymes present. Fermentation broth was added into a final concentration of 5 g/L PASC in 50 mM sodium citrate buffer (pH 5.0) and 100 mM methyl glyoxal (Nacalai Tesque, Inc., Kyoto, Japan); the methyl glyoxal prevents the assimilation of glucose by yeast cells [48]. The reaction was performed at 50 °C for 4 h using a heat block (Thermo Block Rotator SN-06BN; Nissin, Tokyo, Japan) with shaking at 35 rpm, and the supernatant was collected by centrifugation for 10 min at 8000×*g* at 4 °C to remove cells and debris. The amount of glucose in the supernatant was measured by the Glucose CII kit. One unit of PASCase activity was defined as the amount of enzyme needed to produce 1 μmoL of glucose per minute at 50 °C, pH 5.0 (U/mL).

The medium of PASC fermentation was used for the BGL, EG, and CBHI activity assays. The BGL and EG activities were determined as previously described [22]. One unit of the BGL activity was defined as the enzyme amount required for production of 1 μmoL *p*-nitrophenol (*p*NP) in 1 min at 30 °C (U/mL). One unit of EG activity was defined as the absorption at 590 nm of released blue dye in 1 h at 38 °C (U/mL). *p*-Nitrophenyl-*β*-lactopyranoside (*p*NPL, Sigma Co. Ltd, St. Louis, MO, USA) was used for the measurement of CBHI activity as previously described [49]. One unit of CBHI activity was defined as the enzyme amount required for production of 1 μmoL *p*NP in 1 min at 50 °C (U/mL).

## Additional files

10.1186/s13068-015-0344-6 Relative transcription levels of cellulolytic enzyme-encoding genes in recombinant yeast strains. Gene *ACT1* was used as the internal standard. The relative transcription levels were shown normalized to the level observed in strain EG-D-CBHI-D, whose relative transcription level was defined as 1. For each strain, data are presented as the mean ± SD from three independent experiments.
10.1186/s13068-015-0344-6 Time-course profiles of cell growth using host strain BY4741 and recombinant yeast strains in YPD medium. Each strain was inoculated in YPD medium to an initial OD_660_ of 0.05 and then cultured aerobically at 30 °C, 150 rpm for 72 h. For each strain, data are presented as the mean ± SD from three independent experiments.
10.1186/s13068-015-0344-6 PCR primers used in this study.

## Abbreviations

BGL: *β*-glucosidase
EG: endoglucanase
CBH: cellobiohydrolase
CBHI: cellobiohydrolase I
CBHII: cellobiohydrolase II
PASC: phosphoric acid swollen cellulose
CBM: carbohydrate-binding module
CRBF: cell-recycle batch fermentation

## References

[1] Nigam PS, Singh A (2011). Production of liquid biofuels from renewable resources. Prog Energy Combust Sci.

[2] Limayem A, Ricke SC (2012). Lignocellulosic biomass for bioethanol production: current perspectives, potential issues and future prospects. Prog Energy Combust Sci.

[3] Garvey M, Klose H, Fischer R, Lambertz C, Commandeur U (2013). Cellulases for biomass degradation: comparing recombinant cellulase expression platforms. Trends Biotechnol.

[4] Matano Y, Hasunuma T, Kondo A (2012). Display of cellulases on the cell surface of *Saccharomyces cerevisiae* for high yield ethanol production from high-solid lignocellulosic biomass. Bioresour Technol.

[5] Yamada R, Taniguchi N, Tanaka T, Ogino C, Fukuda H, Kondo A (2011). Direct ethanol production from cellulosic materials using a diploid strain of *Saccharomyces cerevisiae* with optimized cellulase expression. Biotechnol Biofuels.

[6] den Haan R, Rose SH, Lynd LR, van Zyl WH (2007). Hydrolysis and fermentation of amorphous cellulose by recombinant *Saccharomyces cerevisiae*. Metab Eng.

[7] Kondo A, Ueda M (2004). Yeast cell-surface display-applications of molecular display. Appl Microbiol Biotechnol.

[8] Bae J, Kuroda K, Ueda M (2015). Proximity effect among cellulose-degrading enzymes displayed on the *Saccharomyces cerevisiae* cell surface. Appl Environ Microbiol.

[9] Hasunuma T, Kondo A (2012). Consolidated bioprocessing and simultaneous saccharification and fermentation of lignocellulose to ethanol with thermotolerant yeast strains. Process Biochem.

[10] Sanda T, Hasunuma T, Matsuda F, Kondo A (2011). Repeated-batch fermentation of lignocellulosic hydrolysate to ethanol using a hybrid *Saccharomyces cerevisiae* strain metabolically engineered for tolerance to acetic and formic acids. Bioresour Technol.

[11] Kondo A, Shigechi H, Abe M, Uyama K, Matsumoto T, Takahashi S, Ueda M, Tanaka A, Kishimoto M, Fukuda H (2002). High-level ethanol production from starch by a flocculent *Saccharomyces cerevisiae* strain displaying cell-surface glucoamylase. Appl Microbiol Biotechnol.

[12] den Haan R, van Rensburg E, Rose SH, Görgens JF, van Zyl WH (2015). Progress and challenges in the engineering of non-cellulolytic microorganisms for consolidated bioprocessing. Curr Opin Biotechnol.

[13] Resch MG, Donohoe BS, Baker JO, Decker SR, Bayer EA, Beckham GT, Himmel ME (2013). Fungal cellulases and complexed cellulosomal enzymes exhibit synergistic mechanisms in cellulose deconstruction. Energy Environ Sci.

[14] Arantes V, Saddler JN (2010). Access to cellulose limits the efficiency of enzymatic hydrolysis: the role of amorphogenesis. Biotechnol Biofuels.

[15] Pack SP, Park K, Yoo YJ (2002). Enhancement of *β*-glucosidase stability and cellobiose-usage using surface-engineered recombinant *Saccharomyces cerevisiae* in ethanol production. Biotechnol Lett.

[16] van Zyl WH, Lynd LR, den Haan R, McBride JE (2007). Consolidated bioprocessing for bioethanol production using *Saccharomyces cerevisiae*. Adv Biochem Eng/Biotechnol.

[17] Hu J, Gourlay K, Arantes V, Van Dyk JS, Pribowo A, Saddler JN (2015). The accessible cellulose surface influences cellulase synergism during the hydrolysis of lignocellulosic substrates. ChemSusChem.

[18] den Haan R, Mcbride JE, Grange DCL, Lynd LR, Van Zyl WH (2007). Functional expression of cellobiohydrolases in *Saccharomyces cerevisiae* towards one-step conversion of cellulose to ethanol. Enzyme Microb Tech.

[19] Takada G, Kawaguchi T, Sumitani J, Arai M (1998). Expression of *Aspergillus aculeatus* No. F-50 cellobiohydrolase I (*cbhI*) and beta-glucosidase 1 (*bgl1*) genes by *Saccharomyces cerevisiae*. Biosci Biotechnol Biochem.

[20] den Haan R, Kroukamp H, van Zyl JH-D, van Zyl WH (2013). Cellobiohydrolase secretion by yeast: current state and prospects for improvement. Process Biochem.

[21] Ilmén M, Den Haan R, Brevnova E, McBride J, Wiswall E, Froehlich A, Koivula A, Voutilainen SP, Siika-Aho M, la Grange DC (2011). High level secretion of cellobiohydrolases by *Saccharomyces cerevisiae*. Biotechnol Biofuels.

[22] Inokuma K, Hasunuma T, Kondo A (2014). Efficient yeast cell-surface display of exo-and endo-cellulase using the *SED1* anchoring region and its original promoter. Biotechnol Biofuels.

[23] Fujita Y, Ito J, Ueda M, Fukuda H, Kondo A (2004). Synergistic saccharification, and direct fermentation to ethanol, of amorphous cellulose by use of an engineered yeast strain codisplaying three types of cellulolytic enzyme. Appl Environ Microbiol.

[24] Andersen N, Johansen KS, Michelsen M, Stenby EH, Krogh KBRM, Olsson L (2008). Hydrolysis of cellulose using mono-component enzymes shows synergy during hydrolysis of phosphoric acid swollen cellulose (PASC), but competition on Avicel. Enzyme Microb Tech.

[25] Bunterngsook B, Eurwilaichitr L, Thamchaipenet A, Champreda V (2015). Binding characteristics and synergistic effects of bacterial expansins on cellulosic and hemicellulosic substrates. Bioresour Technol.

[26] Reyes-Ortiz V, Heins RA, Cheng G, Kim EY, Vernon BC, Elandt RB, Adams PD, Sale KL, Hadi MZ, Simmons BA (2013). Addition of a carbohydrate-binding module enhances cellulase penetration into cellulose substrates. Biotechnol Biofuels.

[27] Shimoi H, Kitagaki H, Ohmori H, Iimura Y, Ito K (1998). Sed1p is a major cell wall protein of *Saccharomyces cerevisiae* in the stationary phase and is involved in lytic enzyme resistance. J Bacteriol.

[28] Yanase S, Yamada R, Kaneko S, Noda H, Hasunuma T, Tanaka T, Ogino C, Fukuda H, Kondo A (2010). Ethanol production from cellulosic materials using cellulase-expressing yeast. Biotechnol J.

[29] Griggs AJ, Stickel JJ, Lischeske JJ (2012). A mechanistic model for enzymatic saccharification of cellulose using continuous distribution kinetics I: depolymerization by EGI and CBHI. Biotechnol Bioeng.

[30] Peckys DB, Mazur P, Gould KL, de Jonge N (2011). Fully hydrated yeast cells imaged with electron microscopy. Biophys J.

[31] Ju X, Grego C, Zhang X (2013). Specific effects of fiber size and fiber swelling on biomass substrate surface area and enzymatic digestibility. Bioresour Technol.

[32] Lynd LR, Weimer PJ, Van Zyl WH, Pretorius IS (2002). Microbial cellulose utilization: fundamentals and biotechnology. Microbiol Mol Biol Rev.

[33] Cruys-Bagger N, Elmerdahl J, Praestgaard E, Tatsumi H, Spodsberg N, Borch K, Westh P (2012). Pre-steady-state kinetics for hydrolysis of insoluble cellulose by cellobiohydrolase Cel7A. J Biol Chem.

[34] Kurasin M, Valjamae P (2011). Processivity of cellobiohydrolases is limited by the substrate. J Biol Chem.

[35] Francisco JA, Stathopoulos C, Warren RA, Kilburn DG, Georgiou G (1993). Specific adhesion and hydrolysis of cellulose by intact Escherichia coli expressing surface anchored cellulase or cellulose binding domains. Bio/Technology.

[36] Fan C, Qi K, Xia X-X, Zhong J-J (2013). Efficient ethanol production from corncob residues by repeated fermentation of an adapted yeast. Bioresour Technol.

[37] Jin M, Gunawan C, Uppugundla N, Balan V, Dale BE (2012). A novel integrated biological process for cellulosic ethanol production featuring high ethanol productivity, enzyme recycling and yeast cells reuse. Energy Environ Sci.

[38] Watanabe I, Miyata N, Ando A, Shiroma R, Tokuyasu K, Nakamura T (2012). Ethanol production by repeated-batch simultaneous saccharification and fermentation (SSF) of alkali-treated rice straw using immobilized *Saccharomyces cerevisiae* cells. Bioresour Technol.

[39] Khaw TS, Katakura Y, Koh J, Kondo A, Ueda M, Shioya S (2006). Evaluation of performance of different surface-engineered yeast strains for direct ethanol production from raw starch. Appl Microbiol Biotechnol.

[40] Matano Y, Hasunuma T, Kondo A (2013). Cell recycle batch fermentation of high-solid lignocellulose using a recombinant cellulase-displaying yeast strain for high yield ethanol production in consolidated bioprocessing. Bioresour Technol.

[41] Brevnova E, McBride J, Wiswall E, Wenger K, Caiazza N, Hau H, Argyros A, Agbogbo F, Rice C, Barret T, Bardsley J, Foster A, Warner A, Mellon M, Skinner R, Shikhare I, Den Haan R, Gandhi C, Bekcher A, Rajgarhia V, Froehlich A, Deleault K, Stonehouse E, Tripathi S, Gosselin J, Chiu YY, Xu H Yeast expressing saccharolytic enzymes for consolidated bioprocessing using starch and cellulose. PCT/US2011/039192(WO/2011/153516). 12-8-2011. 3-6-0110.

[42] McBride JE, Brevnova E, Ghandi C, Mellon M, Froehlich A, Delaault K, Rajgarhia V, Flatt J, Van Zyl WH, Den Haan R, La Grange DC, Rose SH, Penttilä M, Ilmen M, Siika-aho M, Uusitalo J, Hau HH, Rice C, Villari J, Stonehouse EA, Gilbert A, Keating JD, Xu H, Willes D, Shikhare I, Thorngren N, Warner AK, Murphy D Yeast expressing cellulases for simultaneous saccharification and fermentation using cellulose. PCT/US2009/065571. 5-27-2010.

[43] Gibson DG, Young L, Chuang R-Y, Venter JC, Hutchison CA, Smith HO (2009). Enzymatic assembly of DNA molecules up to several hundred kilobases. Nat Methods.

[44] Chen D-C, Yang B-C, Kuo T-T (1992). One-step transformation of yeast in stationary phase. Curr Genet.

[45] Ismail KSK, Sakamoto T, Hatanaka H, Hasunuma T, Kondo A (2013). Gene expression cross-profiling in genetically modified industrial *Saccharomyces cerevisiae* strains during high-temperature ethanol production from xylose. J Biotechnol.

[46] Yamada R, Taniguchi N, Tanaka T, Ogino C, Fukuda H, Kondo A (2010). Cocktail δ-integration: a novel method to construct cellulolytic enzyme expression ratio-optimized yeast strains. Microb Cell Fact.

[47] Yamada R, Nakatani Y, Ogino C, Kondo A (2013). Efficient direct ethanol production from cellulose by cellulase-and cellodextrin transporter-co-expressing *Saccharomyces cerevisiae*. AMB Express.

[48] Kalapos MP (1999). Methylglyoxal in living organisms: chemistry, biochemistry, toxicology and biological implications. Toxicol Lett.

[49] Yamada R, Yoshie T, Sakai S, Wakai S, Asai-Nakashima N, Okazaki F, Ogino C, Hisada H, Tsutsumi H, Hata Y (2015). Effective saccharification of kraft pulp by using a cellulase cocktail prepared from genetically engineered *Aspergillus oryzae*. Biosci Biotechnol Biochem.

[50] Inokuma K, Yoshida T, Ishii J, Hasunuma T, Kondo A (2014). Efficient co-displaying and artificial ratio control of α-amylase and glucoamylase on the yeast cell surface by using combinations of different anchoring domains. Appl Microbiol Biotechnol.

