# Supplementary figures and images for: Combined cell-surface display- and secretion-based strategies for production of cellulosic ethanol with Saccharomyces cerevisiae
Source: Biotechnol Biofuels. 2015 Sep 26;8:162. doi: 10.1186/s13068-015-0344-6 (PMC4584016; doi:10.1186/s13068-015-0344-6)

Figure S1

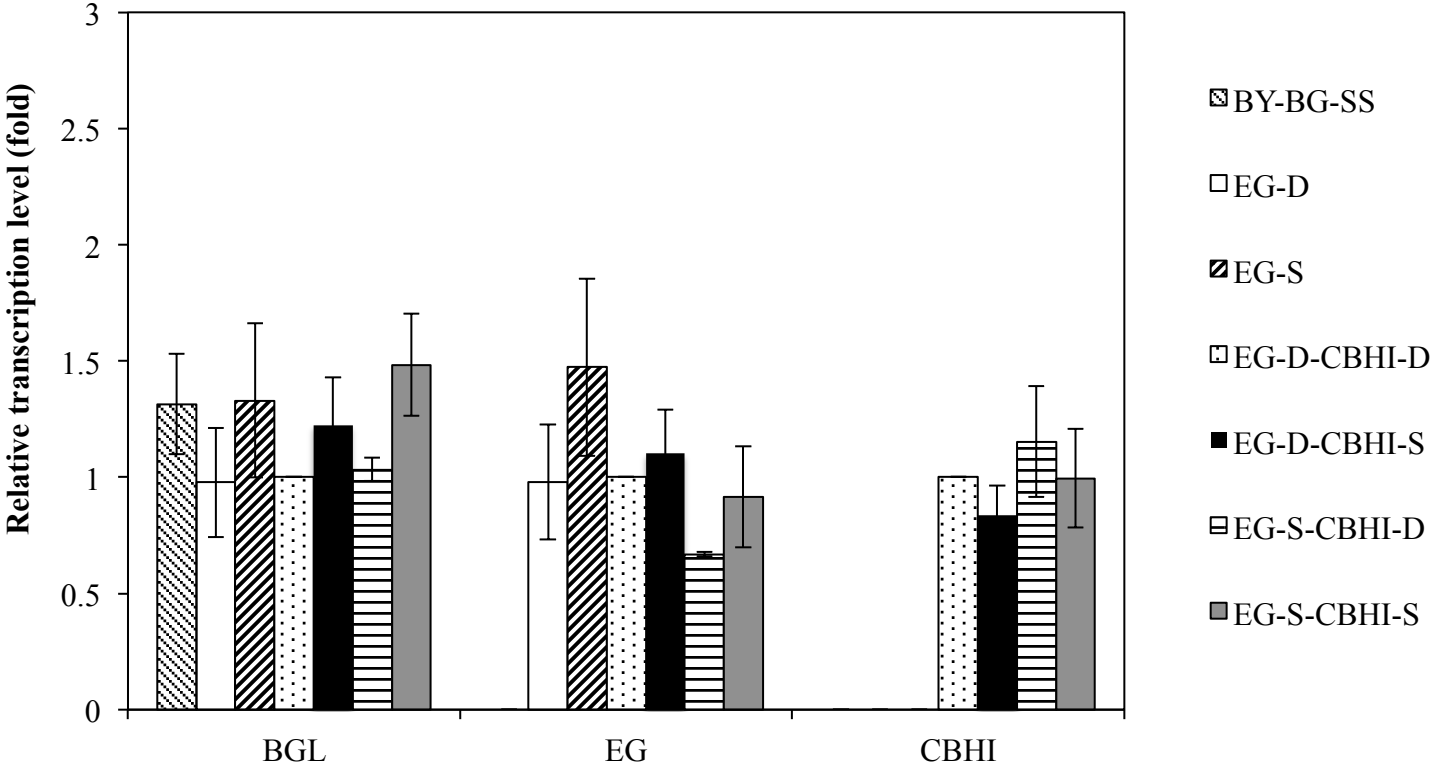

Supplement: Supplementary file 1 — 10.1186/s13068-015-0344-6 Relative transcription levels of cellulolytic enzyme-encoding genes in recombinant yeast strains. Gene ACT1 was used as the internal standard. The relative transcription levels were shown normalized to the level observed in strain EG-D-CBHI-D, whose relative transcription level was defined as 1. For each strain, data are presented as the mean ± SD from three independent experiments. [file 13068_2015_344_MOESM1_ESM.pdf]

## Figure S2

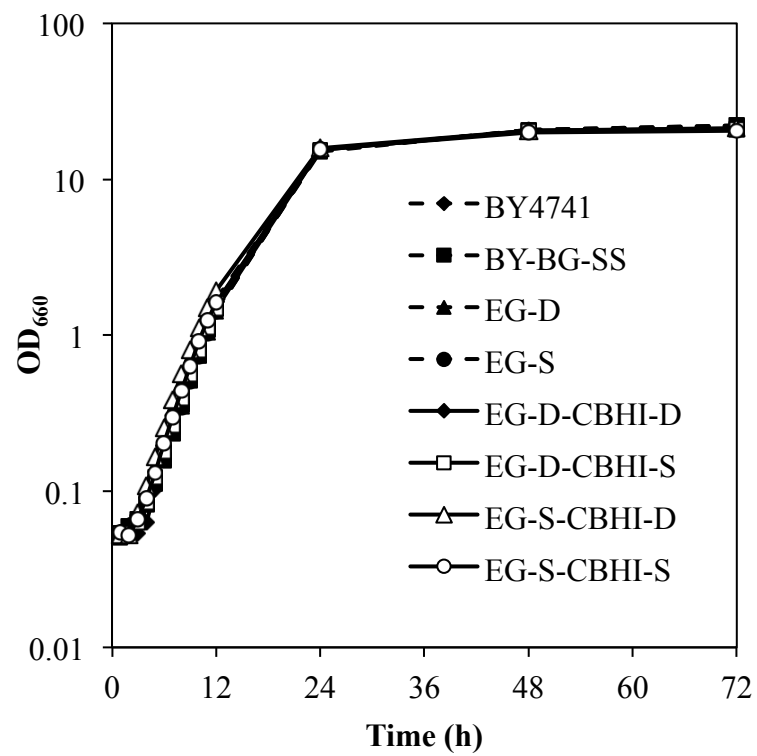

Supplement: Supplementary file 2 — 10.1186/s13068-015-0344-6 Time-course profiles of cell growth using host strain BY4741 and recombinant yeast strains in YPD medium. Each strain was inoculated in YPD medium to an initial OD660 of 0.05 and then cultured aerobically at 30 °C, 150 rpm for 72 h. For each strain, data are presented as the mean ± SD from three independent experiments. [file 13068_2015_344_MOESM2_ESM.pdf]
