# Supplementary material for: Combined cell-surface display- and secretion-based strategies for production of cellulosic ethanol with Saccharomyces cerevisiae
Source: Biotechnol Biofuels. 2015 Sep 26;8:162. doi: 10.1186/s13068-015-0344-6 (PMC4584016; doi:10.1186/s13068-015-0344-6)
Supplement: Supplementary file 3 — 10.1186/s13068-015-0344-6 PCR primers used in this study. [file 13068_2015_344_MOESM3_ESM.pdf]

Table S1

**Primers**	**Sequence (5’-3’)**
TeCBH1-L	GACTTTAATTAAAATGCTAAGAAGAGCTTTACTATTG
TeCBH1-R	GACTGGCGCGCCTTACAAACATTGAGAGTAGTATGGG
TrEG2-F	AATACGTTCGCTCTATTAAGATGAACAAGTCTGTTGCTCCATTG
TrEG2-R	GTTGATAATTTACTCGAGCCTAACTTTCTAGCCAAACATGAAGAAACC
TrEG2-R2	CTCAATGTACTAACTGTACATTATAACTTTCTAGCCAAACATGAAGAAAC
TeCBHI-F	AATACGTTCGCTCTATTAAGATGCTAAAGAAGAGCTTTACTATTGAGC
TeCBHI-R	GTTGATAATTTACTCGAGCCCAAACATTGAGAGTAGTATGGGTTT
TeCBHI-R2	CTCAATGTACTAACTGTACACTACAAACATTGAGAGTAGTATGGGTTT
P-EG2	GGAGCAACAGACTTGTTCATCTTAATAGAGCGAACGTATTTT
EG2-A	CATGTTTGGCTAGAAAGTTAGGCTCGAGTAAATTATCAACTGTCC
EG2-T	GTTTGGCTAGAAAGTTATAATGTACAGTTAGTACATTGAGTCTAAATA
P-CBHI	AGTAAAGCTCTTCTTAGCATCTTAATAGAGCGAACGTATTTT
CBHI-A	CATACTACTCTCAATGTTTGGGCTCGAGTAAATTATCAACTGTCC
CBHI-T	ACTACTCTCAATGTTTGTAGTGTACAGTTAGTACATTGAGTCTAAATA
I2-F	GAAGCCGCGAGTACGAACAATGATG
I2-R	TGGTATTTTCGTGAGCAAACCCAAC
I5-F	CATTGAAGAAGGGAAAGTGGTAACC
I5-R	TCCCTCTCTAATCTGGGTGAGAC
rt-ACT1-F	TGGATTCCGGTGATGGTGTT
rt-ACT1-R	TCAAAATGGCGTGAGGTAGAGA
rt-EG-F	GGTTGTTTGTCTTTGGGTGCTTAC
rt-EG-R	AATTGAGCATTTGTTGGACCACCTT
rt-CBHI-F	CAACTTACTGTCCAGACGACGAAAC
rt-CBHI-R	AAGGAAGAACCAGAGGAGGTAACAC
